# Uncovering the hidden impacts of inequality on mental health: a global study

**DOI:** 10.1038/s41398-018-0148-0

**Published:** 2018-05-18

**Authors:** Shoukai Yu

**Affiliations:** Harvard T. H. Chan School of Public Health, Harvard University, Boston, MA USA

## Abstract

Women are nearly twice as likely as men to suffer from mental illness. This gender disparity in depressive disorders may relate to social inequalities and living standards across nations. Currently, these disparities were not reflected at the level of health policies. This study utilized global data for depressive disorders and socioeconomic data from the United Nations’ World Bank databases and Global Burden of Disease database to demonstrate the correlation between social inequality and gender disparities in mental health. This study investigated the association among the ratio of female to male depressive disorder rates, gross domestic product, the GINI Index, and the gender inequality index for 122 countries. The research yielded some major findings. First, there exists a significant correlation between gender inequality and gender disparities in mental health. Second, the GINI index is significantly associated with male—but not female—depressive disorder rates. Third, gender disparities in depressive disorders are associated with a country’s wealth. These findings can help to inform society, policy-makers, and clinicians to improve the overall health level globally.

## Introduction

According to the World Health Organization (WHO), depressive disorders are major contributors to the world’s health burden; they affect approximately 350 million people worldwide^1–3^. Women are nearly twice as likely as men to suffer from mental illness^4–6^. Although this gender disparity in mental health is reported across diverse geographical regions, societies, populations, and social contexts, there is a dearth of research that explores a link between the impacts of social inequalities and gender disparities on mental health. In this study, the social inequalities include both gender inequality and wealth inequality. Understanding gender disparities in health is very important, according to the National Institutes of Health^7–10^. A growing body of research indicates that psychiatric disorders are largely caused by a combination of stress, environmental, neurobiological, and genetic factors. These poorly understood factors significantly limit the development of effective treatments for these disorders. The major causes for depressive disorders cannot be completely explained by genetic factors^11–13^. The contributions of genetic architectures are difficult to address at the level of health policy. Therefore, attention to social factors, especially with regard to inequality, is critical in approaches to mental health; these factors can be improved dramatically through the implementation of appropriate governmental policies and heightened community awareness.

The brain structure and response to stress are different between females and males^14,15^. For example, community pressure regarding stereotypical social roles based on gender may impact mental health responses differently in women and men^16^. In a male dominated culture, women and men may deal with competition in their workplaces differently. Previous studies also investigate the potential relationship between hegemonic masculinity and mental health in men^17–19^. Human genetic variation exists both within and among populations. These relevant genetic characteristics as well as stress could contribute to gender disparities in mental health^20^. The gender expectations and masculinities may also play an important role in gender disparities in mental health^17,21,22^. In a more general context, gender inequality includes but not limited to domestic violence, sexual abuse, unpaid caring work, higher hours of work, low social status, lack of access to reproductive rights and education^23–27^. Furthermore, the areas related to gender inequality include public health, social work, sociology, and social psychology.

Both gender inequality and wealth inequality have an impact on women’s health at the country level^26,28,29^. For gender inequality research, a series of WHO reports provided in-depth reviews of available literature on the topic of gender equality and mental health in 2000^30^. Since then, there are some studies that have attempted to examine the association between gender inequality and gender disparity in mental health at the country level^31,32^. However, until now the evidence remains inconsistent for the possible impact of gender inequality on gender disparity in mental health^31–34^. In 2007, one study utilized the data from both high income and low and middle income countries and proved that gender equality has no or little impact on the gender disparities in depressive disorders^32^. In 2013, one study, based only on European countries, claimed the potential impact of gender equality on reducing the gender disparity in depressive disorders. Unfortunately, they were unable to provide statistical evidence to prove this association^31^. Therefore, at the global level, the direct statistical evidence to show the association between gender equality and the gender disparity in depressive disorders remains absent.

Wealth inequality has become a frequently and widely discussed topic^31,35–38^. Wealth inequality has impacted general health, including mental health^39–42^. Furthermore, the impact of wealth inequality on mental health has also been investigated^43–45^. Wealth inequality and income inequality are different (Note 1): income represents the money received on a regular basis, while wealth represents the money or properties owed over a lifetime. However, research that attends to gender disparity in depressive disorders and the wealth inequality is limited.

This paper presented the statistical evidence to address this gap in the literature. The WHO has published a series of comprehensive reports about mental health^34,46,47^ and has made a significant effort to collect the data that has permitted an exploration of the gender disparities in mental health^29,30^. The study in this paper captured the impact of social inequality on gender disparities in mental health. Previous studies that have not adequately addressed this problem typically analyzed the data using gender (Notes 2) as a dichotomous variable. Moreover, the scope of many studies has been limited to specific countries^24,48–51^. For example, one study that indicated the potential correlation between the wage gap and gender disparities in mood disorders was limited to the United States and only used the wage gap to measure gender inequality^24^. Another study conducted only in South Korea also indicated that gender inequality might have an impact on mental health. In 2004, one study^27^, conducted in the United Kingdom, indicated domestic violence and abuse toward women related to the greater prevalence of mental illness among women. There is a need to utilize global datasets to identify the impact of inequality on mental health. Unlike existing studies, this study utilized mental health datasets at a global level to conduct the analysis; and the analyses in this study directly focused on the gender disparities on mental health. The novelty of the study in the paper lied in both data integration and the analysis. In order to illustrate the way that the present analysis can be used to better capture the relationships between mental health and inequality, this research also focused specifically on depressive disorders. All of the data were extracted from publicly available datasets and these data represent the largest sample size so far, due to the recent availability of global data on depressive disorders from the Global Burden of Disease database. The novelty analysis was straightforward: the ratio of depressive disorder rates for female to male is used directly as a dependent variable. In this way, gender disparity in depressive disorders can be modeled directly.

A series of statistical models were applied to examine the relationship between gender disparities in mental health and socioeconomic factors. Particular attention was paid to both gender and wealth inequalities. The study aimed to identify whether or not gender disparities in mental health are related to social inequalities, as well as to identify whether or not females respond differently to stress provoked by social inequality as evidenced in mental health outcomes. In this study, social inequality included both wealth inequality and gender inequality. The research was designed to inform public policy as well as to help health professionals reduce gender disparities in mental health and broadly improve mental health outcomes.

## Methods

### Data sources

Mental health data were obtained from the Global Burden of Disease datasets (GBD) website (http://www.healthdata.org/gbd/data, 1 May 2016)^2^. The socioeconomic factors analyzed in this study were the Gender Inequality Index (GII), the GINI Index, and Gross Domestic Product (GDP).The socioeconomic data (GII, GINI Index, and GDP) were obtained from the United Nations’ databases (World Bank and World Economic Forum, 1 May 2016)^52–54^. All datasets were combined by country codes.

### The dependent variable

For mental health data, depressive disorders data, including major depressive disorders and dysthymia, were extracted from the GBD database. In order to obtain the most comprehensive dataset, this study include all clinical case definitions that are consistent with the description of diagnostic criteria for the International Classification of Diseases (ICD)^55^ or Diagnostic and Statistical Manual of Mental Disorders (DSM)^56^. The difference between the diagnostic criteria has been tested and no significant difference has been identified^57^.

In the GBD, the Disability-Adjusted Life Years (DALYs) were calculated by arriving at a sum of the total years of life lost due to premature mortality and the years of life lived with disability to measure health loss based on both mortality and non-fatal health burdens^2,15,46^. The DALY burdens of depressive disorders were obtained from the GBD by country, region, age, and gender for the years 1990, 1995, 2000, 2005, 2010, and 2015. The gender data included the rates of depressive disorders for females, males and both combined. The rates of depressive disorders are referring to the rate per 100,000 of depressive disorders measured by the DALYs. The DALYs combines premature mortality as years of life lost (YLLs) and disability as years lived with disability (YLD)^58,59^. According to WHO^2^, estimates of mood disorders, anxiety disorders, and schizophrenia were calculated and improved with epidemiological evidence and, modified health states and disability weights for GBD databases in 2000s^60,61^.

The first dependent variable in this study is the log-transformed ratio of depressive disorder Rates for Female to Male (log-transformed RRFM) per 100,000. The second dependent variable in this study is DALYs for Depressive Disorder Rates Per 100,000 persons (DDRP) for either of gender.

### The independent variables

The study utilized GII data to determine gender inequality^53^. The GII is a composite measurement of gender inequalities. It measures the loss for women as a result of gender inequality in three areas: reproductive health, empowerment, and the labor market. This index ranges from 0 to 1; the higher value indicates a greater level of inequality. The United Nations Development Programme introduced the GII index in its 2009 and data are available from World Economic Forum Global Gender Gap Index 2014^53,54^.

For socioeconomic data, the GINI Index measures wealth inequality as a distribution of a country’s residents. The index ranges from 0 to 1, and the higher value indicates greater inequality. The GDP measures monetary value of all final goods and services produced in a specific time period. Per capita GDP, in purchasing power parity units may be obtained from World Data Bank. Both GDP and the GINI Index are World development indicators. For the purposes of this study, both the GINI and GDP were obtained in current international currency from the World Bank for the years 1990, 1995, 2000, 2005, 2010, and 2015. The average for those years was calculated for each country. The selection of countries was based on the availability of mental health and socioeconomic data, including GII, the GINI Index, and GDP. The countries with missing data in any of the following categories were removed: depressive disorders, GBD, GII, GINI Index, and GDP.

### Measures

A log-transformed RRFM per 100,000 of depressive disorders was estimated as dependent variable. The independent variables include socioeconomic factors determined by using GII, GINI, and GDP with random intercepts determined for ages and regions. The data preparation included log-transformed RRFM and rescaled socioeconomic indexes by a traditional z-score transformation. The log-transformed RRFM allowed the data to more closely reflect a normal distribution. Similarly, the rescaling of socioeconomic indexes permitted the data to be handled more appropriately for statistical analysis than directly using socioeconomic factors as their original scale, because the original scale of GII and GINI ranges from 0 to 1; while the original scale of GDP ranges from 0 to a real number.

### Control variables

**Fig. 1:**
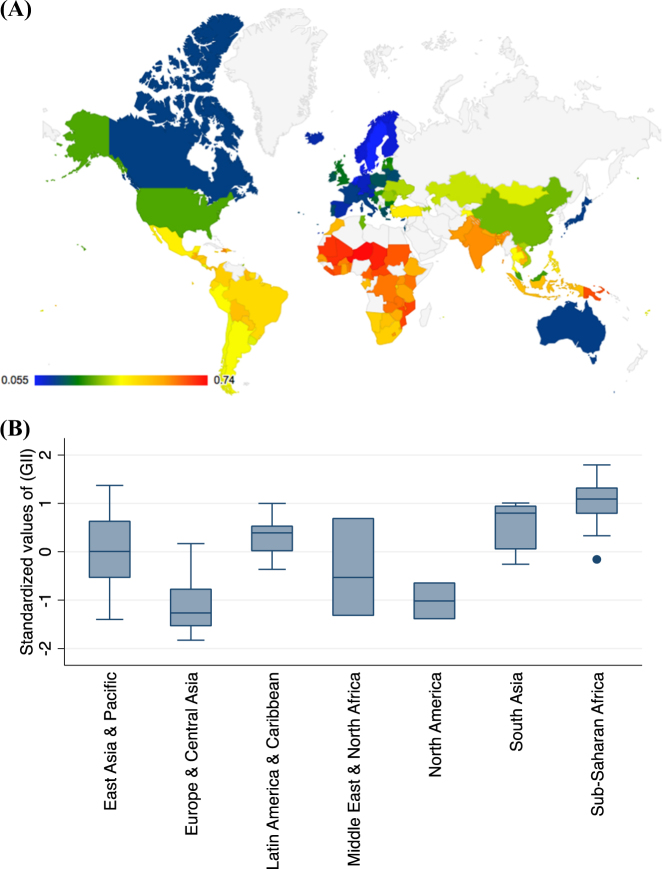
The gender inequality index of countries and seven regions. **a** The map of analyzed countries with their gender inequality index, red color means higher inequality. **b** Gender inequality index for the seven regions, higher values mean higher inequality

The control variables include ages, regions, and years. Age groups were divided into the following categories: under 5 years of age, 5–14 years of age, 15–49 years of age, 50–69 years of age, and 70 years of age or older. Mental health data from 122 countries (Fig. 1) for seven super-regions were included. The super-regions were East Asia & the Pacific, Europe & Central Asia, Latin America & the Caribbean, the Middle East & North Africa, North America, South Asia, and Sub-Saharan Africa (Table 1). The years were 1990, 1995, 2000, 2005, 2010, and 2015.

### Statistical analysis

The mixed models^62^ were fitted using STATA 14^63^. There were six models that were appropriate for the investigation of the relationship between GII and log-transformed RRFM. In the first model, the log-transformed RRFM was estimated as dependent variable. The independent variables include GII, GINI, GDP, and region, with age as a random effect. In the second model, the log-transformed RRFM was estimated as dependent variable. The independent variables include GII, GINI, and GDP, with age, year, and region as random effects. For model 2, the formula is:

$$\begin{array}{ccc}&&{\mathrm{log}}\left( {{\mathrm{relative}}\_{\mathrm{rate}}_{{\it{ijk}}}} \right) = \beta _0 + \beta _1 \times {\mathrm{GII}} + \beta _2 \times {\mathrm{GINI}}\\&& + {\mathrm{\beta }}_{\mathrm{3}} \times {\mathrm{GDP}} + b_i + c_j + d_k,\end{array}$$

where the component relative_rate_*ijk*_ is the relative ratio of female to male mental disorder (Ratio of Rates for Female to Male) as a function of GII, GINI, GDP for region *k*, year *j*, and age *i*. The random effects are age *b*_*i*_, year *c*_*j*_, and region *d*_*k*_. The equation highlights the relationship between relative ratio and gender inequity.

In the third model, the log-transformed RRFM was estimated as dependent variable. The independent variables include GII, GINI, GDP, region, and age, with no random effect variables. The purpose of fitting the third model was to compare the difference of coefficients between the mixed models and a linear regression model. These models highlighted the relationship between the log-transformed RRFM and gender inequality and wealth inequality, with adjustments for GDP. The potential collinearity among predictors was examined using variance inflation factors (VIF). The direct relationship between log-transformed RRFM and GII also was calculated using a Pearson correlation.

Models 4, 5, and 6 were fitted to best identify whether or not the rate per 100,000 of depressive disorders (DDRP) evidenced a direct relationship with any of the socioeconomic factors for females or males. The female DDRP (Model 4), male DDRP (Model 5), and both genders’ DDRP (Model 6) were separately estimated as dependent variable. The independent variables include GII, GINI, GDP, with age and region as the random effects.

## Results

**Fig. 2:**
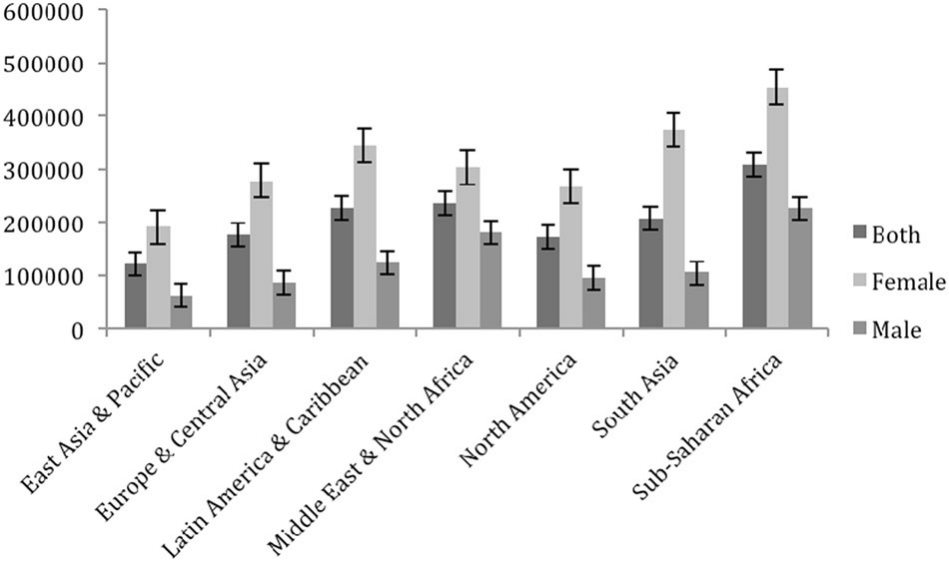
The average depressive disorders for females, males and both genders combined for each region

The depressive disorder rates per 100,000 population (DDRPs) remained relatively stable from 1990 to 2015 for females and males (Fig. 2). For all seven of the super-regions, the mean number of depressive disorders for females was approximately twice that of males, with a range from 1.63 to 3.89. Based on the data from the GBD (Fig. 2), the Sub-Sahara African region had the highest number of depressive disorders for both females (453,705) and males (225,474), whereas the East Asia & Pacific region had the lowest number of depressive disorders for both females (190,818) and males (60,777). The Sub-Sahara African region yielded the highest value, while the regions of Europe & Central Asia the lowest value for gender inequality.

Statistically significant correlations were found between the log-transformed RRFM and GII, as well as between the log-transformed RRFM and GINI index once the mixed effect model was fitted (Table 2). The estimates from the three models were very similar. In Model 1, the VIF score for GII is greater than 2; for Model 2, in which the region effect was considered as a random effect, all of the VIF scores were less than 1.8.

**Table 1:** Number of countries included from each super-region

Region	Number of countries
East Asia & the Pacific	15
Europe & Central Asia	39
Latin America & the Caribbean	23
The Middle East & North Africa	3
North America	2
South Asia	7
Sub-Saharan Africa	33
Sum	122

**Table 2:** Results of statistical analysis between Ratio of Rates for Female to Male (RRFM) and socioeconomic status (GII, GINI, and GDP) for Models 1–3

	Model 1		Model 2		Model 3	
	Exp beta (95% CI)	*P* value	Exp beta (95% CI)	*P* value	Exp beta (95% CI)	*P* value
Intercept (*β*_0_)	1.252(1.157,1.357)	<0.001	1.275 (1.179,1.378)	<0.001	1.072 (1.059,1.085)	<0.001
GII (*β*_1_)	1.043 (1.034,1.053)	<0.001	1.039 (1.032,1.045)	<0.001	1.043 (1.034,1.053)	<0.001
GINI (*β*_2_)	0.976 (0.970,0.982)	<0.001	0.976 (0.971,0.982)	<0.001	0.976 (0.970,0.982)	<0.001
GDP (*β*_3_)	1.038 (1.031,1.045)	<0.001	1.042 (1.033,1.051)	<0.001	1.043 (1.034,1.053)	<0.001

Model 1, RRFM was estimated as dependent variable. The independent variables include GII, GINI, GDP, and region, with age and year as random effects

Model 2, RRFM was estimated as dependent variable. The independent variables include GII, GINI, and GDP, with age, region, and year as random effects

Model 3, RRFM was estimated as dependent variable. The independent variables include GII, GINI, GDP, region, and age, with no random effect variables

There was a significant association (Table 2) between the GII and RRFM. However, no direct associations were found between the GII index and female DDRP and no associations were found between the GII index and the male DDRP (Table 3, Models 4 and 5). Furthermore, there were no associations found between the GII and DDRP for both genders (Model 6 in Table 3). The results together demonstrate that GII index is a hidden factor that correlated with the log-transformed ratio of female to male rates of depressive disorders.

**Table 3:** Results of statistical analysis between Depressive Disorder Rate Per 100,000 population (DDRP)  and socioeconomic status (GII, GINI, and GDP) for Models 4–6

	Model 4		Model 5		Model 6	
	Beta (95% CI)	*P* value	Beta (95% CI)	*P* value	Beta (95% CI)	*P* value
Intercept (*β*_0_)	0.119 (−0.888,1.123)	0.817	−0.217 (−0.990, 0.556)	0.582	−0.042 (−0.936,0.852)	0.926
GII (*β*_1_)	0.039 (0.001, 0.081)	0.061	−0.027 (−0.067, 0.013)	0.180	0.002 (−0.034,0.038)	0.904
GINI (*β*_2_)	−0.013 (−0.039, 0.014)	0.353	0.027 (0.001,0.053)	0.040	0.008 (−0.016, 0.031)	0.531
GDP (*β*_3_)	−0.019 (−0.048,0.011)	0.209	−0.068 (−0.098, −0.039)	0.001	−0.048 (−0.074, −0.021)	0.001

Model 4, female DDRP was estimated as dependent variable. The independent variables include GII, GINI, GDP, with age and region as random effects

Model 5, male DDRP was estimated as dependent variable. The independent variables include GII, GINI, GDP, with age and region as random effects

Model 6, both genders’ DDRP was estimated as dependent variable. The independent variables include GII, GINI, GDP, with age and region as random effects

Interestingly, there were associations between GDP and RRFM (Table 2). Moreover, DDRP for both genders evidenced significant associations with GDP (Table 3; Model 6: −0.048 [−0.074, −0.021], *P*-value < 0.001 for both genders). This shows that societies with higher GDP had lower rates of depressive disorders for both genders.

Greater GII was related to greater RRFM (Relative Ratio (RR) in Table 2 Model 1: 1.043, [1.034, 1.053]; *P*-value < 0.001). However, for the GINI Index, the greater GINI Index at the country level was related to lower RRFM (RR in Table 2 Model 1: 0.976, [0.971, 0.982]; *P*-value < 0.001). For the GDP, there was a significant association between RRFM and GDP. Additionally, the Pearson’s correlation coefficient for RRFM and the GII index were significant (−0.151; *P*-value < 0.001).

## Discussion

This study demonstrated that social inequalities demonstrated a differential impact on mental health for females and males. For GII, greater gender inequality was significant (Model 1: 1.043, [1.034, 1.053]; *P*-value < 0.001) and related to the decreased gender disparity in depressive disorders. This finding strongly suggested that women suffer mentally more than men in societies with greater levels of gender inequality. Combined with the significant correlation between RRFM and the GII index (1.043 [1.034, 1.053], *P*-value < 0.001), gender inequality had a significant impact on gender disparities in depressive disorders. This study provided evidence that social factors, especially gender inequalities may have significant impact on gender disparities in depressive disorders.

This study identified three major findings. First, gender inequality was significantly associated with increased gender disparities in depressive disorders. Previous studies that analyzed depressive disorders separately for females and males failed to detect the association between GII and mental disorder rates that was found here (Table 3)^11^. This study demonstrated that gender inequality may be associated with slightly higher DDRP for females (Model 4: 0.039 [0.001, 0.081], *P*-value = 0.061). Moreover, gender inequality was associated with slightly lower DDRP for males (Model 5: −0.027 [−0.067, 0.013], *P*-value = 0.180). This study identified a significant association (Model 1: 1.043 [1.034, 1.053], *P*-value < 0.001) at the level of ratios, rather than at the level of rates. This distinction permitted an identification of the role that gender inequality played in depressive disorders.

Table 3 provides the information about the relationship between gender inequality and the mental health for female and male separately. For female, the estimate 0.039 [0.001, 0.081] is larger than 0, which indicates the greater gender inequality is related to the greater depression rate for women. While for male, the estimate −0.027 [−0.067, 0.013] is less than 0, which indicates the greater gender inequality is related to the lower depression rate for men. Both estimates do not reach the significance level for p values, while the *P* value for the ratio of female rates to male rates is significant (Table 2). This is also one of the reasons why this association between gender inequality and mental health is hidden. Gender inequality includes but not limited to domestic violence, sexual abuse, unpaid caring work, higher hours of work, low social status, lack of access to reproductive rights and education^23–27^. The stress responses have been linked to depression^43,64^. In a male dominated culture, women and men may deal with competition in their workplaces differently. Previous studies also investigate the potential relationship between hegemonic masculinity and depressive disorders in men^17–19^.

Second, men suffered from more mental health problems than women when dealing with situations of high wealth inequality (Models 4 and 5). This finding challenged assumptions that females would prove more emotionally or mentally sensitive to many social inequalities^65,66^. However, a high GINI index was significantly associated with high DDRP for males (Model 5: 0.027 [0.001, 0.053], *P*-value < 0.05), whereas a high GINI index is not associated with high DDRP for females (Model 4: −0.013 [−0.039, 0.014], *P*-value = 0.353). This result is noteworthy and expands upon the contributions made by a recent study^24^ that indicated that the wage gap may be related to higher rates of major depression for females in the United States. One possible explanation could be that males are more mentally sensitive to wealth inequality, due to either stress or their genetic makeup^20,33,67,68^. From a biological point of view, the presence of the Y chromosome and different hormones could also contribute to brain reactions to the wealth inequality. Yet, stereotypical social roles could put pressure on men to excel in the work place, producing greater levels of stress in men. This possibility would reaffirm the need to address inequality as an integral part of a plan to improve mental health among males. The higher GINI index was significantly associated with lower RRFM (Table 2, Models 1–3). However, the decreased gender disparity in depressive disorders was due to an increased DDRP for males, as opposed to a lower rate of depressive disorder rate among females.

Third, the GDP showed a direct association with RRFM, after adjusting for other socioeconomic factors and regional effects. Yet, the higher GDP correlate with slightly higher RRFM. Moreover, GDP did correlate with the prevalence of depressive disorders for both genders (Model 6). This finding would suggest that higher overall wealth level for a country is not related to reducing gender disparity in depressive disorders. However, improving the overall level of wealth may indeed reduce the prevalence of depression in a specific population^69–71^.

In addition to this work’s three major findings, there was one other finding that merits mention. Different geographical locations showed different regional impacts on gender disparities associated with depressive disorders (Fig. 1). This finding was consistent with those from previous studies^57,72,73^. These results indicate that regional or geographical effects, as well as genetic factors (population differentiation, human genetic variation for different human populations), potentially played a role in gender disparities in depressive disorders. Regional and geographical variations could be due to the combination of effects of cultural, environmental, and socioeconomic factors.

There is substantial variability existed in GII index between countries (Fig. 1). Similarly, there are also substantial variability existed in GINI index (Supplementary Figure 1) and GDP (Supplementary Figure 2) between countries. The high wealth inequality countries tend to cluster at Latin America and Caribbean, and some countries in the south part of Sub-Saharan Africa. The countries with higher GDP tend to have lower GINI index, such as Canada, the USA, Australia, and countries in Europe. Furthermore, there are some developing countries, such as China and some countries in the north part of Sub-Saharan Africa, although the GDP is not very high, the wealth inequality index is relatively low, which demonstrate the indirect correlation between GDP and GINI index. Overall, there is a cluster tendency for all of the three independent variables. Compared to the other two independent variables, the cluster tendency for GDP index is stronger.

## Conclusion

This is one of the first studies to successfully provide statistical evidence of an association between gender disparities in psychiatric disorders and social inequalities at a global level. These results contribute to the growing evidence that social inequality has an independent effect on population-specific depressive disorders^24,48^. This study was enhanced by a multi-faceted approach to the matter of inequality that utilized both the United Nations’ definition of inequality and measures of inequality such that gender inequality could be captured more precisely. The novelty in the paper lied in the analysis using existing databases. The overall results suggested that diverse aspects of social inequality, including both gender inequality and wealth inequality, evidenced differential impacts on mental health for both genders.

Caution should be exercised in interpreting and extrapolating the study results to posit broader generalizations regarding mental health. The study results only demonstrated correlations rather than causal links between inequality and depressive disorders. A focus on causal relationships between policies, such as economic, education and public health and mental health may not adequately capture the complexity of social interactions and the nature of mental disorders. The causal relationship could be further explored from the genomics and etiology aspects. Moreover, this study analyzed gender inequality and wealth inequality at the country level, and there is no apparent correlation between GII and GINI indexes. If future analysis is utilized for research on a local scale, such as at the level of community or county, the correlation between gender inequality and wealth inequality should be taken into account in the modeling process. Furthermore, attention should be drawn to the potential collinearity between the independent variables. Additionally, this study was based solely on the genders recorded in the GBD database (female, male, and both combined), with no information on lesbian, gay, bisexual, and transgender populations.

Improvement in a given population’s mental health would require a multidisciplinary policy approach that addresses socioeconomic determinants of health. Wealth inequality has become a pressing issue in a wide range of countries internationally^23,74–76^. Moreover, many researchers have shown that socioeconomic status has impacted general health^39–42^. Recently, many studies have focused on the gender differences regarding health^77–80^. Unlike most previous studies on inequality and health, this research specifically demonstrated the association between the effects of socioeconomic inequality gender disparities on mental health. Future research could further explore the causal relationships that might exist between social factors and mental health outcomes. Currently, the global burden of disease database lack country level data for mental health for majority countries^81^. The data at the country level for the global burden of disease study could further improve our understanding the association between socioeconomic determinants and mental health.

The findings presented here provided strong evidence of a relationship between high gender inequality and a higher ratio of depressive disorder rates for both females and males. This significant correlation might be partially explained by gender discrimination. Gender prejudice, either overt or covert, could subject females to the experience of greater barriers to accessing community resources, including mental health care, that contribute to better health. The regions that exhibited high rates of common mental disorders also exhibited high levels of inequality, as reported by the WHO^6^. The United Nations emphasized the need for increased attention to factors that link gender disparities to health, including education, inclusion in policy decisions, participation, income, and differential socioeconomic status in its 17 sustainable development goals. It would be important to focus on the impact of policies designed to further equality, including both gender equality and wealth equality, in order to address existing mental health disparities and achieve the highest possible level of health for all people.

### Notes

1. The definitions of income inequality and wealth inequality are different. However, wealth and income inequality usually are not distinguished by their original definitions in the existing literature on the GINI index. Therefore, in this paper, wealth inequality is used to denote all wealth and income equalities, unless the inequality is only measured by income. Then the phrase income inequality will be used.
2. According to the WHO, gender refers to the range of socially constructed roles and characteristics of women and men; sex refers to biological differences^82,83^. The aim of this study is to emphasize the impact of both gender inequity and socioeconomic inequality on mental health at the country level. Moreover, the causes of depressive disorders are related to combined social and biological effects. Therefore, in this paper, the word gender is used to denote all sex and gender differences, unless those differences can be fully attributed to biological differences. Then the word sex will be used.
3. For socioeconomic data, the GDP measures monetary value of all final goods and services produced in a specific time period. Per capita GDP, in purchasing power parity units may be obtained from World Data Bank. The GINI Index measures wealth inequality as a distribution of a country’s residents. The index ranges from 0 to 1, and the higher value indicates greater inequality. Both GDP and the GINI Index are World development indicators.
4. The GII is a composite measurement of gender inequalities. It measures the loss for women as a result of gender inequality in three areas: reproductive health, empowerment, and the labor market. This index ranges from 0 to 1; the higher value indicates a greater level of inequality. The United Nations Development Programme introduced the GII index in its 2009 and data are available from World Economic Forum Global Gender Gap Index 2014 for 141 countries^53,54^.

## Electronic supplementary material

Supplementary Material
