# Supplementary material for: Uncovering the hidden impacts of inequality on mental health: a global study
Source: Transl Psychiatry. 2018 May 18;8:98. doi: 10.1038/s41398-018-0148-0 (PMC5959880; doi:10.1038/s41398-018-0148-0)
Supplement: Supplementary file 1 — Supplementary Material [file 41398_2018_148_MOESM1_ESM.docx]

**Supplementary material**

**Supplementary Figure S1.** The map of analyzed countries with their GINI index, red color means higher GINI index.

**Supplementary Figure S2.** The map of analyzed countries with their GDP, red color means higher GDP.

**Supplementary Figure S3.** Time series of the numbers depressive disorders for female, male and both genders for seven regions. The year points include 1990, 1995, 2000, 2005, 2010, and 2013. The purple lines represent the number depressive disorders for female, blue lines represent the number depressive disorders for male and green lines are for both gender combined.


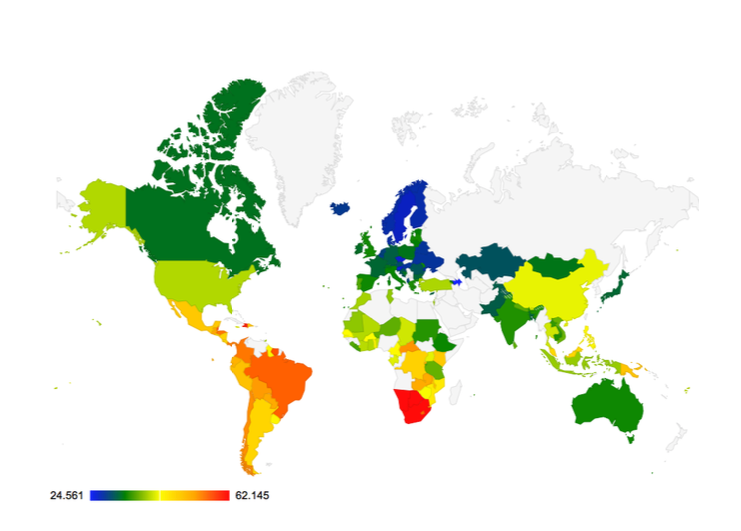


**Supplementary Figure S1**. The map of analyzed countries with their GINI index, red color means higher GINI index.


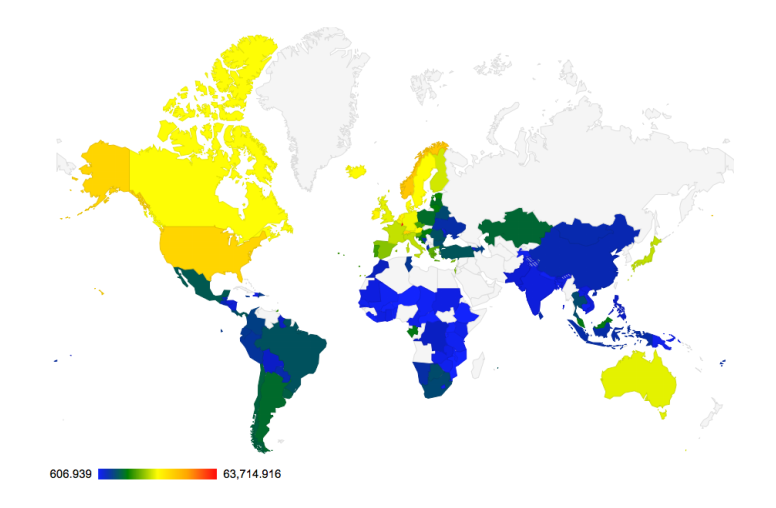


**Supplementary Figure S2**. The map of analyzed countries with their GDP, red color means higher GDP.

**Supplementary Figure S3**. Time series of the numbers depressive disorders for female, male and both genders for seven regions. The year points include 1990, 1995, 2000, 2005, 2010, and 2013. The purple lines represent the number depressive disorders for female, blue lines represent the number depressive disorders for male and green lines are for both gender combined.
